# Whole-Genome Sequence of *Aeromonas hydrophila* Strain AH-1 (Serotype O11)

**DOI:** 10.1128/genomeA.00920-16

**Published:** 2016-09-01

**Authors:** Gabriel Forn-Cuní, Juan M. Tomás, Susana Merino

**Affiliations:** Departamento de Genética, Microbiología y Estadística, Sección Microbiologia, Virología y Biotecnología, Facultad de Biología, Universidad de Barcelona, Barcelona, Spain

## Abstract

*Aeromonas hydrophila* is an emerging pathogen of aquatic and terrestrial animals, including humans. Here, we report the whole-genome sequence of the septicemic *A. hydrophila* AH-1 strain, belonging to the serotype O11, and the first mesophilic *Aeromonas* with surface layer (S-layer) to be sequenced.

## GENOME ANNOUNCEMENT

*Aeromonas hydrophila* is a water-borne opportunistic pathogen of poikilothermic animals, including fish, reptiles, and mammals (1). Although it is an integral part of the intestinal flora of healthy fish (2), some *A. hydrophila* strains can cause severe outbreaks of motile aeromonad septicemia (MAS), causing huge economic losses in the aquaculture industry (3). In humans, *A. hydrophila* pathogenesis involves gastrointestinal and wound infections and even septicemia in immunocompromised patients (4). *Aeromonas* strains are considered a contaminant agent by the U.S. Environmental Protection Agency and are routinely monitored in drinking water. The virulence of *A. hydrophila* is multifactorial and has been associated with several pathogenic factors, including, but not limited to, surface polysaccharides, surface layers (S-layers), secretion systems, and flagella (5).

S-layers are bacterial cell surface proteins associated with different pathogenic functions, such as cell adhesion, antigenic properties, and protection against host complement system lysis and phagocytes (6). *A. hydrophila* AH-1 is a septicemic strain and the first mesophilic *A. hydrophila* strain with an S-layer to be sequenced to date. The analysis of the genome from this strain will allow in-depth understanding of the evolution and lateral transfer of genes involved in the S-layer production in bacteria and its importance in virulence and pathogenicity.

The genome of *A. hydrophila* AH-1 strain was fully sequenced using Illumina MiSeq II, generating a total of 6,856,223 paired reads with 82× coverage. Read quality analysis and trimming were done with Prinseq 0.20.4 (7). *De novo* assembly with SPAdes 3.6.0 (8) resulted in 218 scaffolds larger than 500 kb.

Genome annotation was performed both via the NCBI Prokaryotic Genome Annotation Pipeline (PGAAP) and Rapid Annotations using Subsystems Technology (RAST). The complete genome of *A. hydrophila* AH-1 is 5,123,179 bp, with 60.9% G+C content, and codes for 4,773 predicted genes, eight rRNAs, and 95 tRNA sequences.

### Accession number(s).

This whole-genome shotgun project has been deposited at DDBJ/EMBL/GenBank under the accession no. LYXN00000000 (BioProject PRJNA323709). The version described in this paper is the first version, LYXN01000000.
